# Viral Aetiology of Central Nervous System Infections in Adults Admitted to a Tertiary Referral Hospital in Southern Vietnam over 12 Years

**DOI:** 10.1371/journal.pntd.0003127

**Published:** 2014-08-28

**Authors:** Le Van Tan, Le Hong Thai, Nguyen Hoan Phu, Ho Dang Trung Nghia, Ly Van Chuong, Dinh Xuan Sinh, Nguyen Duy Phong, Nguyen Thi Hoang Mai, Dinh Nguyen Huy Man, Vo Minh Hien, Nguyen Thanh Vinh, Jeremy Day, Nguyen Van Vinh Chau, Tran Tinh Hien, Jeremy Farrar, Menno D. de Jong, Guy Thwaites, H. Rogier van Doorn, Tran Thi Hong Chau

**Affiliations:** 1 Oxford University Clinical Research Unit, Ho Chi Minh City, Vietnam; 2 Hospital for Tropical Diseases, Ho Chi Minh City, Vietnam; 3 Pham Ngoc Thach University of Medicine, Ho Chi Minh City, Vietnam; 4 Centre for Tropical Medicine, Nuffield Department of Medicine, University of Oxford, Oxford, United Kingdom; 5 Department of Medical Microbiology, Academic Medical, Center, University of Amsterdam, Amsterdam, The Netherlands; Duke-NUS, Singapore

## Abstract

**Background:**

Central nervous system (CNS) infections are important diseases in both children and adults worldwide. The spectrum of infections is broad, encompassing bacterial/aseptic meningitis and encephalitis. Viruses are regarded as the most common causes of encephalitis and aseptic meningitis. Better understanding of the viral causes of the diseases is of public health importance, in order to better inform immunization policy, and may influence clinical management.

**Methodology/Principal Findings:**

Study was conducted at the Hospital for Tropical Diseases in Ho Chi Minh City, a primary, secondary, and tertiary referral hospital for all southern provinces of Vietnam. Between December 1996 and May 2008, patients with CNS infections of presumed viral origin were enrolled. Laboratory diagnostics consisted of molecular and serological tests targeted at 14 meningitis/encephalitis-associated viruses.

Of 291 enrolled patients, fatal outcome and neurological sequelae were recorded in 10% (28/291) and 27% (78/291), respectively. Mortality was especially high (9/19, 47%) amongst those with confirmed herpes simplex encephalitis which is attributed to the limited availability of intravenous acyclovir/valacyclovir. Japanese encephalitis virus, dengue virus, herpes simplex virus, and enteroviruses were the most common viruses detected, responsible for 36 (12%), 19 (6.5%), 19 (6.5%) and 8 (2.7%) respectively, followed by rubella virus (6, 2%), varicella zoster virus (5, 1.7%), mumps virus (2, 0.7%), cytomegalovirus (1, 0.3%), and rabies virus (1, 0.3%).

**Conclusions/Significance:**

Viral infections of the CNS in adults in Vietnam are associated with high morbidity and mortality. Despite extensive laboratory testing, 68% of the patients remain undiagnosed. Together with our previous reports, the data confirm that Japanese encephalitis virus, dengue virus, herpes simplex virus, and enteroviruses are the leading identified causes of CNS viral infections in Vietnam, suggest that the majority of morbidity/mortality amongst patients with a confirmed/probable diagnosis is preventable by adequate vaccination/treatment, and are therefore of public health significance.

## Author Summary

Central nervous system (CNS) infections are important diseases worldwide. The spectrum of infections is broad, encompassing bacterial/aseptic meningitis and encephalitis. Viruses are regarded as the most common causes of encephalitis and aseptic meningitis. Better understanding of the causes of the diseases is of public health importance, in order to better inform immunization policy, and influence clinical management. We describe the clinical features and infectious causes of 291 adults with clinically suspected CNS infections of presumed viral origin. We show that CNS viral infections in Vietnam are associated with high morbidity and mortality. Mortality was especially high (47%) amongst those with herpes simplex encephalitis which is attributed to the limited availability specific antiviral drugs in our setting. Japanese encephalitis virus, dengue viruses, herpes simplex virus and enteroviruses were the most common viruses detected, followed by rubella virus, varicella zoster virus, mumps virus, cytomegalovirus, and rabies virus. Our study represents the broadest yet investigation of the possible viral causes of the CNS infections in adults in Vietnam, with a diagnostic yield of 32%. The results show that the majority of morbidity/mortality amongst patients with a confirmed/probable diagnosis could be prevented by adequate vaccination or treatment, and are therefore of public health significance.

## Introduction

Central nervous system (CNS) infections are important diseases worldwide. The spectrum of infections is broad, encompassing bacterial meningitis, aseptic meningitis and encephalitis. The estimated incidence of encephalitis worldwide is between 3.5 and 7.4 cases per 100,000 person years [1]. While the clinical course of viral meningitis and encephalitis may overlap, viral meningitis is usually self-limiting [2], whereas the mortality from viral encephalitis ranges from 4.6% to 29% [3]–[8] and nearly 50% of survivors have persistent neurological sequelae after 6 months follow up [7]. Viruses are regarded as the most common causes of encephalitis and aseptic meningitis, and the specific viral aetiology of the diseases is diverse and dependent on geographical, temporal, host-immunity and age factors [3], [7]–[10]. The aetiology is in part driven by the introduction of immunization programs and/or the (re)emergence of (new) pathogens.

In Southeast Asia, in 1998 Nipah virus emerged in Malaysia and Singapore [11], [12] and spread to Bangladesh where it causes annual outbreaks of fatal encephalitis [13]. Over the last 16 years, enterovirus 71 has emerged and caused large outbreaks of hand foot and mouth disease, sometimes associated with fatal encephalitis in young children. Likewise, Japanese encephalitis virus (JEV) is a leading cause of encephalitis in children and occasionally in young adults in many countries in Asia including Vietnam [9], [14]–[16]. In Vietnam, JEV vaccine was first introduced in 1997, and had been administered to all children 1–5 years of age in 437 (65%) of 676 districts by 2007 [14]. By 2008, 91% of the target population in Vietnam had received JEV vaccination [17].

Studies in Western countries have revealed that herpes simplex virus (HSV) varicella-zoster virus (VZV) and enteroviruses (EVs) are the leading causes of encephalitis/aseptic meningitis in adults [4], [7], [18], whereas in two recent prospective descriptive studies in Vietnam JEV, dengue virus (DENV), HSV, EVs and VZV were frequently detected in adults CNS infections of presumed viral origin [9], [19]. Of note, in these two studies virus diagnostic tests were limited to these 5 types, possibly underestimating the aetiological diversity of the infections. Better understanding of the causes of the diseases is of public health importance, in order to better inform immunization policy, and may influence clinical management. Herein we report the results of a 12 year study investigating the clinical and laboratory features of 291 HIV uninfected adults with presumed viral CNS infections admitted to a tertiary referral hospital in southern Vietnam.

## Materials and Methods

### Setting

The study was conducted at an infectious disease ward of the Hospital for Tropical Diseases (HTD) in Ho Chi Minh City, a primary, secondary, and tertiary referral hospital for infectious diseases in both children and adults for all southern provinces of Vietnam. The hospital has 550 beds, 35,000 admissions annually, and serves a population of 42 million people. Any adult with severe CNS infections in southern Vietnam is referred to HTD. The patients who present to this hospital are therefore representative of the whole of southern Vietnam. Patient enrolment started in December 1996 and is on-going. The present study reports findings of the 291 consecutive patients enrolled between December 1996 and May 2008.

### Patient inclusion criteria

All adult patients (age ≥15 years) presenting with clinically suspected CNS infections of presumed viral origin, based on the clinical judgment of admitting physicians, negative HIV serology, and with no evidence of purulent bacterial, eosinophilic, cryptococcal and tuberculous meningitis by CSF cell count, culture and/or microscopy, were eligible to enter the study.

### Clinical data and specimen collection

Detailed demographic and clinical data, including routine blood and CSF haematology and biochemistry were collected on case record forms at enrollment and during hospitalization. Clinical outcomes were assessed at discharge using the Glasgow Outcome Scale and was defined as death, full recovery (no abnormality), and severe (greatly affecting function, i.e. dependence), moderate (deficit affecting function but not dependence), or minor (abnormality detectable but not affecting function) neurological sequelae, based on neurological examination, degree of independent functioning and controllability of seizures [20]. For microbiological investigations, acute CSF and serum were collected at enrolment.

### Routine microbiological tests

As part of routine care, CSF specimens of the enrolled patients were cultured and/or examined by microscopy for detection of bacterial/*C. neoformans*/*M. tuberculosis* infection with the use of standard methods if clinically indicated. *Eosinophilic* meningitis was diagnosed by CSF examination, and defined by the presence of more than 10 eosinophils per ml of CSF or >10% of total CSF white cells. All patients were tested for antibodies to HIV.

### Clinical management

Between 1997 and 2004, oral acyclovir 4 g per day was given if herpes simplex encephalitis (HSE) was suspected because intravenous (IV) acyclovir was not available in our hospital at that time. From 2005, patients with suspected HSE were either given oral valacyclovir 3 g per day or oral acyclovir 4 g per day. IV acyclovir 1.5 g per day was only given to patients who could afford to pay for their medications pending HSV PCR results. Irrespective of the aetiology, supportive therapy for patients with encephalitis of presumed viral infection was an important cornerstone of management. Seizures were controlled with IV benzodiazepines, phenytoin or phenobarbital, and where necessary sedation and mechanical ventilation. Medical management of raised intracranial pressure included elevating the head of bed, IV mannitol, and intubation with mechanical hyperventilation. Careful attention was paid to the maintenance of respiration, cardiac rhythm, fluid and electrolyte balance, prevention of deep vein thrombosis, aspiration pneumonia, and secondary bacterial infections.

### Aetiological investigations

#### Molecular diagnostic analyses

All tests were performed retrospectively on aliquots of whole CSF stored at −80°C immediately after lumbar puncture. Total nucleic acid (NA) was extracted from 100 µl of CSF specimens using an automated easyMAG system (bioMérieux, Marcy l'Étoile, France), following the manufacturer's instructions. Internally controlled real-time (RT-) PCRs were utilized to detect several meningitis/encephalitis-associated viruses [including HSV, cytomegalovirus (CMV), VZV, Epstein-Barr Virus (EBV), EVs, Nipah virus, influenza A and B virus, flaviviruses (generic) and Mumps virus] as well as four bacterial pathogens (including *Streptococcus pneumoniae*, *Neisseria meningitidis*, *Haemophilus influenzae* type b and *Streptococcus suis*). If clinically indicated, saliva was collected and tested by rabies PCR. All the PCR procedures were carried out as described in our previous studies [8], [9], [21], [22].

#### Serology

A capture IgM ELISA assay (Venture Technologies, Sarawak, Malaysia) that utilizes inactivated antigens from JEV and DENV1–DENV4 was used to detect and distinguish between JEV- and DENV-specific IgM antibodies in CSF, and was performed as previously described [23].

A chemiluminescent microparticle immunoassay (CMIA, Abbott diagnostic division, Lake Forest, Illinois 60045, USA) was used for the detection of IgM antibodies to rubella virus in serum, and was performed according to the manufacturer's instructions.

For detection of EBV antibodies in serum, an IgG/IgM ELISA (Enzygnost, Dade-Behring, Marburg, Germany) using recombinant viral capsid antigen (VCA) and EBV nuclear antigen (EBNA) was used, and was performed according to the manufacturer's instructions.

Detection of CMV-specific IgM and IgG antibodies in sera of patients that had CMV DNA detected in CSF samples were done with use of Abbott Architect CMV IgM/IgG assays (Abbott diagnostic division), and was performed according to the manufacturer's instructions.

#### Sequencing

For identification of specific flaviviral species, flavivirus PCR amplicons were sequenced using the Big Dye Terminator v1.1 Cycle Sequencing Kit (Applied Biosystems, Carlsbad, CA, USA), following the manufacturer's instructions.

### Diagnostic interpretation

Patients were categorised as having confirmed, probable or possible diagnoses, and no aetiological agent found (see Table 1). A confirmed diagnosis was established if viral nucleic acid or viral specific IgM was detected in CSF for JEV, DENV, HSV, EVs, mumps and VZV. In some instances, the detection of the virus in CSF or in other body fluids alone is insufficient and requires further supporting evidence for interpretation of the results. Details are presented in Table 1.

**Table 1:** Diagnostic interpretation of laboratory tests performed.

Virus	Evidence by detection of	Other supporting evidence	Diagnostic interpretation
JEV	IgM in CSF		Confirmed
DENV	IgM or viral RNA in CSF		Confirmed
HSV	Viral DNA in CSF		Confirmed
EVs	Viral RNA in CSF		Confirmed
Rubella#	IgM in serum	Rash suggestive of rubella	Probable
Mumps	Viral RNA in CSF		Confirmed
CMV	Viral DNA in CSF	IgG and IgM detected in acute blood$	Probable
CMV	Viral DNA in CSF	IgG detected in acute blood without IgM¥	Possible
CMV	Viral DNA in CSF	No acute blood available for serologic testing	Possible
VZV	Viral DNA in CSF		Confirmed
EBV	Viral DNA in CSF	VCA IgG and EBNA IgG detected in acute blood without VCA IgM¥	Possible
EBV	Viral DNA in CSF	No acute blood available for serologic testing	Possible
Rabies*	Viral RNA in saliva		Confirmed

# only patients with rash suggestive of rubella were tested;

$ suggestive of recent/acute infection;

¥ suggestive of past infection [44]; For both viruses encephalitis/encephalomyelitis/(poly)radiculitis may be (rare) manifestations of acute infection or of reactivation associated with severe (iatrogenic) immunocompromise. By demonstrating presence of EBNA IgG for EBV and CMV IgG for CMV in the absence of IgM acute infection can be ruled out. All patients were HIV negative, but information about signs or symptoms of severe immunocompromise were unfortunately not available, and considered as possible cause as virus reactivation cannot be ruled out;

*rabies PCR was only performed on collected saliva of patients with a clinical syndrome suggestive of rabies (e.g. hydrophobia, hypersalivation and aerophobia).

### Statistical analysis

Chi-square test, Fisher's exact test, independent samples t test and the Wilcoxon rank-sum test were used to compare data between groups of patients when appropriate by using either SPSS for Windows version 14 (SPSS Inc, Chicago IL, USA) or statistical software R version 2.9.0 (http://www.r-project.org).

### Ethics

For the period between 1996 and October 2007, the samples analysed were anonymized, residual CSF and blood specimens that were collected as part of routine care. The use of these clinical specimens for the purpose of improving knowledge about the causative agents of CNS infections in Vietnam was approved by the Scientific and Ethical Committee of the Hospital for Tropical Diseases, Vietnam.

From November 2007 onward, the prospective study of patients was approved by the Scientific and Ethical Committee of the Hospital for Tropical Diseases, Vietnam and the Oxford University Tropical Research Ethics Committee, UK, OxTREC number: 004-07. Physicians entering patients into the study were responsible for obtaining their written informed consent from the patient. If the patient was unconscious, the written informed consent was obtained from a relative or a family member.

## Results

### Patients, characteristics and clinical outcome

Between December 1996 and May 2008, 1601 patients with CNS infections were admitted to the infectious ward of the HTD, of whom 291 fulfilling the entry criteria of CNS infections of presumed viral origin were enrolled. 190 (65%) were male, and 235 (89%) were referred from other hospitals. The median duration of hospital stay was 10 days [interquartile range (IQR): 7–18]. A total of 71 (25%) patients received acyclovir or valacyclovir: oral acyclovir (n = 39), IV acyclovir (n = 2) and oral valacyclovir (n = 30) (Table 2).

**Table 2:** Patient characteristics and comparisons between patients with or without a confirmed viral aetiology.

Patient characteristics	Whole group (n = 291)	Unknown/possible causes (n = 192)$	Confirmed/probable viral aetiology (n = 93)	Diag. vs. undiag. *P* value (OR; 95%CI)
Demographics				
Male	190 (65)	125 (65)	61 (66)	1 (1.02; 0.59–1.78)
Age	25 (19–34)	26 (20–35)	22 (17–32)	0.005
Referral from other hospital	235 (89)	151 (88)	78 (92)	0.62 (1.21; 0.55–2.67)
Hospitalization (days)	10 (7–18)	10 (7–20)	9 (6–15)	0.005
Symptom				
Day of illness	6 (3–8)	6 (4–9)	5 (3–7)	0.13
History of fever	263 (91)	168 (88)	90 (99)	0.002 (12.85; 1.71–96.60)
Vomiting	143 (53)	86 (48)	54 (62)	0.028 (1.78; 1.06–3.01)
Headache	234 (82)	148 (79)	82 (90)	0.023 (2.40: 1.10–5.20)
Convulsion	77 (29)	57 (32)	19 (24)	0.19 (0.64; 0.33–1.21)
Sign				
Fever at admission	181 (62)	118 (62)	61 (66)	0.51 (1.19; 0.69–2.08)
Neck stiffness	162 (65)	103 (64)	56 (68)	0.48 (1.23; 0.68–2.27)
Cranial nerve palsies	17 (6)	11 (6)	6 (7)	0.79 (1.14; 0.33–3.52)
Skin rash	15 (5)	8 (4)	6 (7)	0.39 (1.58; 0.44–5.39)
GCS≤9	83 (29)	62 (32)	21 (23)	0.09 (0.61; 0.32–1.11)
GCS = 15 and No focal Neurological signs	75 (28)	51 (27)	24(28)	0.89(0.59; 1.04–1.85)
Laboratory				
CSF WC (cells/mm^3^)	93 (24–247)	58 (12–176)	180 (82–371)	<0.001
CSF protein (g/l)	0.8 (0.48–1.12)	0.75(0.4–1.1)	0.87(0.57–1.19)	0.31
CSF lactate (mmol/l)	2.3 (1.8–2.89)	2.3(1.8–2.9)	2.4(1.8–2.7)	0.97
CSF/blood glucose	0.6 (0.52–0.68)	0.62 (0.52–0.71)	0.59(0.53–0.66)	0.20
Antiviral treatment	71 (24)	46(24)	25(27)	0.66(1.16; 0.63–2.12)
Acyclovir	41*	24(12.5)	18(19)	
Valacyclovir	30	22(11.5)	7(8)	
Outcome at discharge				
Death	28 (10)	15 (8)	13 (14)	0.48
Severe sequelae	49 (17)	33 (17)	15 (16)	
Moderate sequelae	17 (6)	11 (6)	6 (7)	
Mild sequelae	12 (4)	9 (5)	2 (2)	
Full recovery#	183 (63)	122 (64)	57 (61)	

**Note**: Data are Number (%) of patients, denominators may vary slightly; continuous variables were reported as median, interquartile range; Diag: diagnosed, undiag: undiagnosed;

$ the 6 patients with bacterial DNA detected in CSF alone or together with EBV DNA were not included in this patient group. As comparison found not statistical difference in clinical outcomes and patient characteristics between patients with possible viral diagnoses and those with no diagnoses (data not shown) except for CSF WCC: median 170 cells/mm^3^ (IQR 70–305) vs. 54 (IQR 11–160), respectively (*P* = 0.03), clinical data of these two groups were combined and compared against patient group of definitive or probable viral diagnosis;

*of whom one was treated with intravenous acyclovir on admission and one had oral acyclovir 5 days after admission before being treated with intravenous acyclovir.

# full recovery was based on clinical evaluation at hospital discharge and did not include cognitive assessment, learning, etc.

A low Glasgow coma score (≤9) was recorded in 82 (29%) of the patients on admission. A fatal outcome was recorded in 28 (10%) patients; and 78 (27%) patients suffered neurological sequelae at discharge, which was severe in 49 (17%), moderate in 17 (6%) and mild in 12 (4%). There were no differences in outcome detected between patients who had confirmed/probable causes identified versus those with possible/no aetiological agent identified (Table 2).

The patient's characteristics, clinical outcome and distribution of the enrolled patients over the study period are presented in Table 2.

### Clinical samples available for aetiology investigations

Virus and bacterial specific PCR investigations were performed on CSF specimens of all 291 enrolled patients, while serologic tests for IgM antibodies against DENV and JEV were done in 278 patients due to insufficient volumes of CSF from the remaining patients (Table 3). Rabies PCR was performed on saliva of 1 patient with a clinical syndrome suggestive of rabies. Serologic testing for IgM antibodies to rubella virus was performed on sera of six patients with a non-confluent maculopapular rash, suggestive of rubella on admission. Further testing for the presence of antibodies to EBV (VCA IgM, VCA IgG and EBNA IgG) and CMV (IgM and IgG) were done on available acute blood samples from 22/24 and 4/5 patients that had viral DNA detected in CSF by EBV and CMV PCRs, respectively (Table 3).

**Table 3:** Comparisons between characteristics of patients with HSV- and other pathogens infection.

Patient characteristics	Other pathogens (n = 74)*	JEV (n = 36)	DENV (n = 19)	EVs (n = 8)	HSV (n = 19)	HSV vs. other pathogens *P* value (OR; 95%CI)	HSV vs. JEV *P* value (OR; 95%CI)	HSV vs. DENV *P* value (OR; 95%CI)	HSV vs. EVs *P* value (OR; 95%CI)
Demographics									
Male	46(62)	24 (67)	12 (63)	7 (88)	15 (79)	0.27(2.28;0.69–7.57)	0.34(1.88; 0.51–6.9)	0.27(2.27;0.52–9.99)	1(0.58;0.05–6.4)
Age	20.5(17–30)	18 (16–22)	28 (17–38)	23 (19–32)	32 (21–41)	0.001	<0.001	0.26	1.36
Referral from other hospital	62(93)	32 (89)	16 (84)	8 (100)	14 (86)	0.63(0.65;0.11–3.64)	1(0.75; 0.11–4.95)	0.49(0.5;0.3–0.72)	1(0.75;0.58–0.97)
Hospitalization (days)	9(6–14)	8 (6–16)	9 (6–13)	4 (3–6.5)	9 (4–22)	0.9	0.85	0.84	0.03
Symptom									
Day of illness	5(3–6)	5 (4–6.8)	5 (3–9)	2.5 (2–4.5)	5 (7–9)	0.013	0.029	0.46	0.001
History of fever	71(98)	35 (100)	17 (94)	8 (100)	19 (100)	1(0.79;0.71–0.88)	-	0.49(0.46;0.32–0.68)	-
Vomiting	45(62)	28 (78)	9 (50)	5 (71)	9 (60)	0.91(0.93;0.3–2.9)	0.31(0.42;0.12–1.57)	0.59(1.5;0.34–6.53)	1(0.6;0.08–4.17)
Headache	65(90)	32 (91)	17 (94)	8 (100)	17 (90)	1(0.92;0.17–4.8)	1(0.79;0.12–5.24)	1(0.5;0.04–6.12)	1(0.69;0.53–0.91)
Convulsion	12(18)	5 (16)	5 (33)	7 (100)	7 (47)	0.038(3.94;1.2–13)	0.038(4.55;1.23–18.35)	0.43(1.88;0.39–9.01)	0.051(1.87;1.17–3.01)
Sign									
Fever at admission	44(60)	24 (67)	9 (47)	5 (63)	17 (90)	0.014(5.8;125–27)	0.13(4.15;0.84–21.49)	0.004(10.71;1.84–62.45)	0.18(6.8;0.5–91.45)
Neck stiffness	45(66)	25 (76)	8 (50)	5 (63)	11 (79)	0.53(1.87;0.48–7.39)	1(1.17;0.26–5.28)	0.1(3.81;0.74–19.66)	0.57(2.2;0.24–20.4)
Cranial nerve palsies	4(6)	3 (9)	1 (5)	0	2 (13)	0.39(2.36;0.4–14.16)	0.64(1.52;0.23–10.15)	0.58(2.5;0.2–31)	1(1.46;1.08–1.98)
Skin rash	6(8)	1 (3)	2 (11)	0	0	0.34(1.28;1.15–1.48)	1(1.53;1.27–1.88)	0.49(2.1;1.48–3.085)	-
GCS≤9	14(19)	6 (17)	5 (26)	0	7 (37)	0.13(2.5;0.83–7.5)	0.12(2.92;0.81–10.49)	0.45(1.77;0.39–7.93)	0.13(1.6;1.13–2.36)
Laboratory									
CSF WC (cells/mm^3^)	200(85–420)	222 (120–430)	160 (20–288)	420 (88–1091)	123 (54–223)	0.18	0.038	0.81	0.09
CSF protein (g/l)	0.91(0.57–1.27)	0.91(0.6–1.18)	0.8 (0.52–1.03)	0.58(0.41–1.07)	0.76(0.57–0.96	0.3	0.16	0.77	0.62
CSF lactate (mmol/l)	2.45(2.2–2.88)	2.05(1.7–2.55)	2.3 (1.6–2.4)	2.45 (2.33–2.65)	2.45(2.22–2.78)	0.11	0.024	0.15	0.87
CSF/blood glucose	0.59(0.55–0.68)	0.59(0.53–0.64)	0.6 (0.52–0.65)	0.56(0.50–0.58)	0.59(0.56–0.68)	0.35	3.75	0.62	0.056
Antiviral treatment	16(22)	8(22)	6(32)	0	9(47)¥	0.024(3.26;1.13–9.39)	0.07(3.15;0.95–10.41)	0.27(2.23;0.52–10)	0.027(1.78;1.15–2.74)
Acyclovir	10(14)	5(14)	4(21)		8(42)				
Valacyclovir	6(8)	3(8)	2(11)		1(5)				
Outcome at discharge									
Death	4(5)	2 (6)	1 (5)	0	9 (47)	<0.001	0.002	0.001	0.027
Severe sequelae	11(15)	7 (19)	3 (16)	0	4 (21)				
Moderate sequelae	5(7)	3 (8)	1 (5)	0	1 (5)				
Mild sequelae	2(3)	0	1 (5)	1 (13)	0				
Full recovery	52(70)	24 (67)	13 (68)	7 (88)	5 (26)				

**Note**: Data are Number (%) of patients, denominators may vary slightly; continuous variables were reported as median, interquartile range;

*Not including 19 HSV patients;

¥ Outcome was not fully evaluated in one patient because of being transferred to other hospital.

### Confirmed/probable viral aetiology

We found confirmed/probable viral infection in 93 (32%) patients (see Table 4). JEV was the most common virus detected (n = 36, 12%) followed by DENV (n = 19, 6.5% - CSF serology, n = 17 and CSF PCR, n = 2), HSV (n = 19, 6.5%) and EVs (n = 8, 2.7%) (Table 4).

**Table 4:** Frequency of specific viruses detected and dual infections.

Virus	Number of tested CSF samples	Other specimen Type (n)	Method used	Detected in (%)	Diagnostic category$
JEV	278		Serology (IgM)	36 (12.4)	Confirmed
DENV	278		Serology (IgM)/flavivirusPCR+sequencing	19 (6.5)	Confirmed
HSV	291		PCR	19 (6.5)	Confirmed
EBV	291	Serum (22)	CSF PCR+serology (IgM+IgG)	22 (8.2)	Possible
			CSF PCR	2 (0.7)#	Possible
EVs	291		PCR	8 (2.7)	Confirmed
Rubella		Serum (6)	Serology (IgM)	6	Probable
Mumps	291		PCR	2 (0.7)	Confirmed
CMV	291	Serum (1)	CSF PCR+serology (IgM+IgG)	1 (0.3)	Probable
		Serum (3)	CSF PCR+serology	3 (1)	Possible
			CSF PCR	1 (0.3)#	Possible
VZV	291		PCR	5 (1.7)	Confirmed
Rabies		Saliva (1)	PCR	1 (0.3)	Confirmed
Influenza A virus	291		PCR	0	–
Influenza B virus	291		PCR	0	–
Nipah virus	291		PCR	0	–
Dual infections				23 (8%)	–
EBV-JEV				6	–
EBV-DENV				2	–
EBV-HSV				1	–
EBV-VZV				1	–
EBV-*S. pneumoniae* *				2	–
CMV-JEV				2	–
CMV- Mumps				1	–
CMV- EVs				1	–
HSV-DENV				2	–
HSV-EVs				1	–
DENV-Rubella				1	–
JEV-*S. pneumoniae*				1	–
Mumps-*S. suis* serotype 2				1	–
*S. suis*-*N. meningitidis*				1	–
Total confirmed/probable viral diagnosis				93 (32%)	

**Note**:

$ interpretation of the results is in accordance with criteria provided in Table 1;

# no sera available for serologic tests;

*clinical outcome and associated characteristics of all patients in who bacterial DNA was detected in CSF by PCR are detailed in Appendix Table 1.

Characteristics and clinical outcome of patients with or without a confirmed/probable viral aetiology, and patients with JEV, DENV, EVs and HSV infections, are presented in Tables 2, 3 and Table 5 (with additional data for DENV patients). Overall, the characteristics of patients with or without a confirmed/probable viral aetiology were similar, although patients with a confirmed/probable viral aetiology were significantly younger, had higher frequency of history of fever, vomiting and headache on admission, and higher CSF white blood cell counts, but they required a shorter hospital stay (p<0.05 for all).

**Table 5:** Characteristics of patients that had DENV RNA/IgM antibody detected in CSF.

Patient	Age (y)	Gender (M/F)	Illness day	Rash	HCT	PLT	SGOT	SGPT	Vomiting	Headache	Convulsion	Fever	Neck stiffness	Cranial nerve palsies	GCS	Outcome$	WCC	Neu (%)	Lymph (%)	Lactate (mmol/l)	CSF/blood glucose	Protein (g/l)	DENV diag.	Other Path detected
1	30	F	26	N	44	160000	NA	NA	Y	Y	N	N	N	Y	15	5	120	12	88	4.4	0.49	2.1	IgM CSF	EBV CSF PCR
2	32	M	3	N	41	388000	60	62	N	Y	N	N	Y	N	15	5	200	18	82	2.4	0.65	0.54	IgM CSF	N
3	25	M	8	N	49	NA	52	142	N	Y	NA	N	Y	N	15	5	310	64	36	2.4	0.77	1.06	IgM CSF	N
4	39	M	30	N	41	232000	56	60	Y	Y	N	N	NA	N	15	5	86	7	93	1.5	0.48	0.45	IgM CSF	N
5	25	M	22	N	43	NA	NA	NA	N	Y	N	N	N	N	15	5	173	1	99	3.4	0.42	0.9	IgM CSF	EBV CSF PCR
6	31	M	3	N	42	NA	NA	NA	Y	Y	N	Y	N	NA	15	2	354	4	96	1.5	0.64	1	IgM CSF	N
7	38	M	6	N	44	197000	NA	NA	N	Y	Y	Y	Y	N	12	5	600	75	25	1.8	0.63	0.94	IgM CSF	N
8	15	F	5	N	49	387000	NA	NA	Y	Y	N	N	Y	N	13	5	160	8	92	2.9	0.76	0.26	IgM CSF	N
9	17	F	3	N	34	147000	28	35	N	Y	Y	Y	N	N	7	4	252	8	92	2.2	0.55	1.51	IgM CSF	N
10	21	M	3	N	45	186000	41	29	Y	Y	Y	Y	Y	N	10	5	288	7	93	2.4	0.60	1.45	IgM CSF	HSV CSF PCR
11	42	F	4	N	39	182000	62	71	N	Y	N	N	N	N	8	2	4	70	30	4	0.72	0.6	RNA CSF	N
12	36	M	9	N	43	131000	27	104	Y	Y	NA	Y	NA	N	7	1	36	0	100	2.4	0.58	0.7	IgM CSF	HSV CSF PCR
13	15	M	2	Y*	36	120000	41	25	NA	NA	NA	Y	Y	N	7	5	188	4	96	1.6	0.55	0.8	IgM CSF	RUBELLA IgM
14	15	F	9	Y#	36	167000	42	22	Y	Y	Y	Y	N	N	9	5	49	15	43	2.3	0.63	0.5	IgM CSF	N
15	67	F	2	N	39	300000	32	40	Y	Y	NA	Y	N	N	15	3	4	NA	NA	2.4	0.48	0.4	RNA CSF	N
16	28	M	14	N	38	389000	49	91	Y	Y	N	Y	Y	N	14	2	20	25	75	0.9	0.58	0.2	IgM CSF	N
17	38	M	6	N	37	384000	24	104	N	Y	N	N	NA	N	15	5	20	10	90	1.3	0.64	0.75	IgM CSF	N
18	16	F	3	N	34	369000	NA	NA	N	Y	N	N	N	N	15	5	365	4	89	2	0.48	1.43	IgM CSF	N
19	22	M	5	N	43	130000	263	193	N	N	Y	N	Y	N	14	5	58	4	76	1.3	0.70	0.94	IgM CSF	N

Note: y: years; M: male, F: female; HCT: haematocrit; PLT: platelet; SGOT: serum glutamic oxaloacetic transaminase; SGPT: serum glutamic-pyruvic transaminase; GCS: Glasgow coma score; WCC: white blood cell; Neu: neutrophils; Lymph: lymphocyte; NA: not available, Y: yes; N: No;

*non-confluent maculopapular rash suggestive of rubella infection,

# details not recorded;

$ 1: Death; 2: Severe sequelae; 3: Moderate sequelae; 4: Mild sequelae; 5: Full recovery; patients with high platelet count (>150000 per microliter) are in bold.

The incidence of death (47%) and neurological sequelae (26%) by discharge in the 19 patients with HSV encephalitis (HSE) was significantly higher than that of patients with other infections (Table 3). Nine (47%) patients with HSE received acyclovir/valacyclovir; in one of whom the discharge outcome was not fully assessed because of being transferred to another hospital. There was a trend towards better outcome amongst those who received drug earlier. The median days of illness before acyclovir/valacycolovir administration was 3 days (range 3–5) in survivors compared to 7 days (range 5–13) (P = 0.05) in those who died.

Patients infected with JEV were mostly young adults and were significantly younger than the other patients: median age 18 years (IQR 16–22) compared to 28 years (IQR 17–38; P = 0.02) for those with DENV and 32 years, (IQR 21–41; P<0.001) for those with HSE.

### Possible viral aetiology

A possible viral cause was detected in 28 (10%) patients. EBV DNA was detected by PCR in CSF of 24 patients (Table 4). Among these, evidence of past EBV infection was seen in 22/22 (100%) tested patients (VCA IgG and EBNA IgG positive) but none had evidence of acute infection (negative VCA IgM) (Table 4). Similarly, CMV DNA was detected in the CSF of 5 patients of whom 4 had acute sera available for serologic tests. All had detectable CMV specific IgG, but three had undetectable IgM (Table 4).

### Bacterial diagnosis by PCR investigation

Of 291 patients fulfilling the inclusion criteria of CNS viral infections, evidence of bacterial infection by PCR analysis of CSF was found in 8 (2.7%), including 5 with *S. pneumoniae*, 2 with *S. suis* serotype 2, and one with PCR positive for both *S. suis* and *N. meningitidis*. Three of the 8 bacterial PCR positive patients were treated with antibiotics prior to admission. Clinical outcomes and CSF laboratory data of these patients are detailed in Supplementary Table S1.

### Dual infections

Twenty-three patients (8%) had tests that were positive for more than one agent (Table 4). In the majority (16/23, 70%) of these, the CSF was PCR positive for EBV or CMV plus another agent. In those with positive CSF EBV PCR, 6 were positive for JEV, 2 with DENV, and 1 each with HSV, VZV and *S. pneumoniae*, respectively. In those with positive CSF CMV PCR, 2 were also positive for JEV (n = 2), and 1 each with mumps and EVs, respectively. Among EBV patients there was no statistical difference in viral load (as suggested by the obtained Ct values) between patients with or without other positive results (median Ct value 37, range 34–40 vs. 35, range 32–41, respectively, P = 0.19).

### Time to death

Twenty-eight (10%) patients died during hospitalization. 60% died within the first two weeks of illness, and 93% died within the first two weeks of hospital admission.

### Monthly distribution

**Figure 1:**
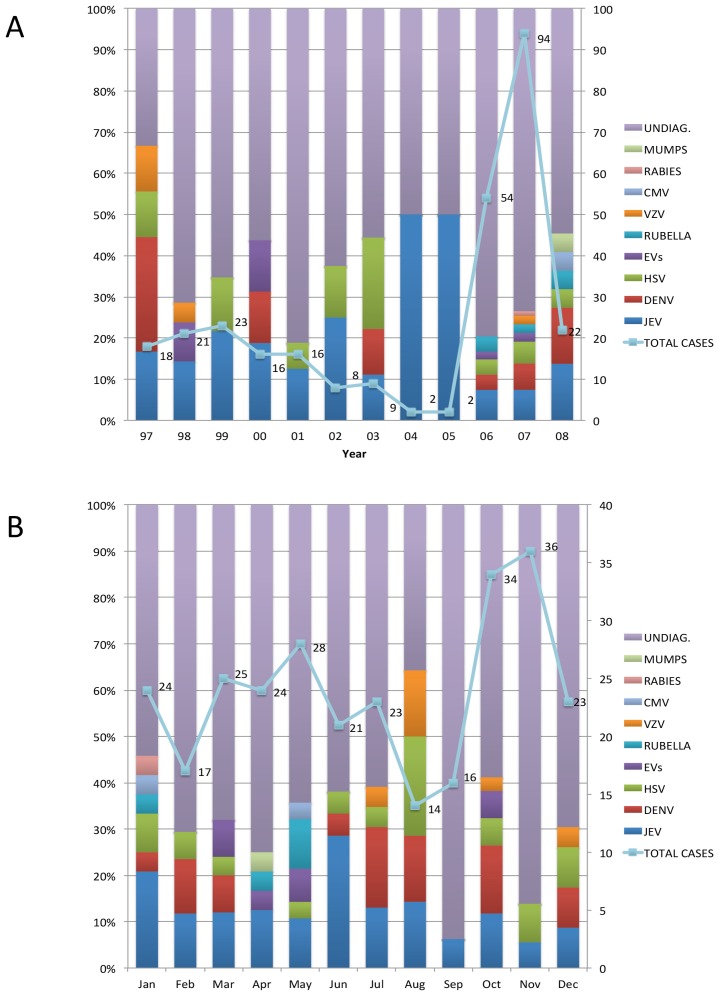
Distribution of total enrolled patients and confirmed/probable causes over the study period A) over year and B) over month; bars showing the diagnostic yields (%) and lines representing the patient number.

Two hundred and ninety one patients were enrolled over a 12-year period (Figure 1A). Encephalitis cases distributed throughout the year with a slight peak in October and November. Similarly, there is no clear seasonal trend for specific viruses except for JEV cases, which peaked in June (Figure 1B), when the southern part of Vietnam enters the rainy season.

## Discussion

We describe the clinical features and infectious aetiology of 291 adults with suspected CNS infections of presumed viral origin admitted to a tertiary referral hospital in southern Vietnam over 12 years. The incidence of death in this study was high at 10% (28/291). And this was particularly marked in patients with confirmed HSE (9/19, 47%). The mortality from viral encephalitis reported in other studies ranged from 4.6% to 20% [3]–[7]. Studies assessing outcome from HSE have reported mortality ranging from 5% in France [4], to 11% in the UK [7] and 18% in the USA [3]. High-dose IV acyclovir is recommended for the treatment of HSE [24], but is often unavailable or unaffordable in resource-poor countries, such as Vietnam. The limited availability of acyclovir/valacyclovir and the lack of its empirical prescription may explain the high mortality from HSE in our study. According to a recent study conducted in our hospital, oral valacyclovir, which is considerably cheaper, may be an acceptable early treatment for suspected HSE in resource limited settings [25].

Neurological sequelae were also frequent in our patients, affecting 27% (78/291). More subtle impairments of cognitive function may have been missed in our assessment; a recent study performed in the UK found 45% of patients to have persistent neurological sequelae 6 months after diagnosis [7]. The lack of specialist rehabilitation services in less well-resourced countries makes the problems of neurological sequelae particularly serious for patients and their families.

The diagnostic yield of 32% of the present study is in accordance with previous findings (16–52%) [3]–[7], [18], and further illustrates the big challenge to establish a confirmed infectious aetiology in patients with acute CNS infections. Together with our previous reports, the data confirm that JEV, DENV, HSV and EVs are the leading causes of CNS viral infections in Vietnam [8], [9], [19], and highlights much-needed efforts for national vaccination campaigns against vaccine preventable diseases due to viruses as JEV, rubella and rabies in Vietnam.

As of 2008, which is at the end of this report, it was recorded that JEV vaccine was administered to 91% of the target population in Vietnam [17]. Because, data on vaccination status of the patients was not available, it remains unknown whether the JEV patients in the present study had received (sufficient doses of) JE vaccine. Of note, in our previous study in children with viral encephalitis in southern Vietnam in 2004, of 191 enrolled children, only 19.5% had received at least one dose of JE vaccine. [8]. Follow-up study is therefore needed to assess the effect of this immunization campaign on the overall incidence of JEV in Vietnam.

Currently, Vietnam is amongst the countries that have yet to include rubella vaccination in their routine immunization programmes [26], and has experienced notable outbreaks between 2005–2007 with ≥3000 cases/year, and ∼800 cases in 2008 [27]. We may have underestimated the number of cases of encephalitis due to rubella in our study, since only patients with rashes where the diagnosis was suspected were tested. Likewise, rabies is responsible 100 cases per year annually in Vietnam. Rabies control can be achieved through vaccination of humans and animals, dogs in particular [28]–[30]. Rabies control in Vietnam is challenging because of limited public awareness (particularly among pet owners), large numbers of stray dogs, and the lack of a national vaccination program [30]–[32].

Neurological manifestations of DENV infection have been recorded in about 0.5–20% and 4–47% of patients admitted with classical dengue and encephalitis-like illness, respectively in endemic areas [33]. Similarly, the 19 DENV patients were all admitted with clinical sing/symptom of acute CNS infection without a typical picture of classical dengue infection. Although currently there are no standardised case definitions or diagnostic criteria available for this clinical entity [33], [34], according to criteria recently proposed by Carod-Artal et al. [33], 15/19 (79%) dengue patients in our study can be classified as having dengue encephalitis.

While the biological significance of the detection of EBV/CMV DNA in CSF remains unknown, the high frequency of EBV/CMV DNA detection in CSF together with other potential CNS pathogens observed in this study confirms the findings of previous reports [35]–[38]. Past infection was also documented in 22/22 and 3/4 EBV and CMV patients, respectively. The detection of EBV/CMV DNA in CSF of these patients may be a result of the inflammatory processes and white blood cell recruitment leading to CSF entry of EBV/CMV infected cells. However, it cannot be ruled out that under certain circumstances (e.g. co-infection with another CNS pathogen) the virus may reactivate and cause or aggravate CNS infection [39]–[41].

Evidence of bacterial infection was detected in 2% of patients with negative CSF Gram stains and culture and with clinical and laboratory data compatible with CNS viral infections, suggesting that PCR should always be considered to exclude CNS infections in patients with treatable bacterial meningitis, particularly in countries as Vietnam where antibiotics are frequently prescribed in community and hospital settings prior to presentation at a facility where a definitive microbiological diagnosis could be made [42].

Our study has some limitations. First, this is a hospital-based descriptive study and patient admission to the research ward is biased by the availability of beds at the time the patients are admitted to the hospital. This in part, explains the fluctuation in patient numbers enrolled over the study period (Figure 1A). Therefore the data may not closely represent for the wider community. Second, we did not look for all potential infectious causes of CNS infections in Vietnam (including measles virus [43]), or for non-infectious (immune or endocrine) causes. Third, diagnostics by IgM testing of acute samples might be suboptimal (e.g. in case of JEV and DENV) both in terms of sensitivity and specificity. Fourth, samples from other body compartments such as rectal and throat swabs were not collected for aetiological investigation in this study (e.g. in case of enterovirus infection), which could have increased the total diagnostic yield [8]. Fifth, undiagnosed cases could be caused by novel pathogens which would have gone undetected. However, this study represents the broadest investigation yet of the possible viral causes of the CNS infections in adults in Vietnam, with a diagnostic yield of 32%. The results suggest that the majority of morbidity/mortality amongst patients with a confirmed/probable viral CNS infection is preventable by adequate vaccination and/or treatment, and are therefore of public health significance.

## Supporting Information

Checklist S1 STROBE checklist.
(DOC)

Table S1 Characteristics of patients positive for bacterial PCRs.
(DOCX)
