# Supplementary material for: Viral Aetiology of Central Nervous System Infections in Adults Admitted to a Tertiary Referral Hospital in Southern Vietnam over 12 Years
Source: PLoS Negl Trop Dis. 2014 Aug 28;8(8):e3127. doi: 10.1371/journal.pntd.0003127 (PMC4148224; doi:10.1371/journal.pntd.0003127)
Supplement: Table S1 — Characteristics of patients positive for bacterial PCRs. (DOCX) [file pntd.0003127.s002.docx]

**Supplementary Table S1:** characteristics of patients positive for bacterial PCRs

| Patient | Illness day | Add. From other hos.* | CSF WCC | CSF  Neu (%) | CSF  Lymphocyte  (%) | CSF Lactate  (mmol/L) | CSF: blood glucose ratio | CSF Protein (g/L) | CSF Appearance | Antibiotic use (Pre-admission) | Antibiotic use (Duration) | Outcome | Bacterial PCR  (Ct value) | Viral  Diagnosis  (Ct value) |
| --- | --- | --- | --- | --- | --- | --- | --- | --- | --- | --- | --- | --- | --- | --- |
| 1 | 4 | Y | 180 | 30 | 70 | 3.1 | 0.54 | 0.92 | Clear | N |  | Death | SP (38) | JEV |
| 2 | 7 | Y | 285 | 8 | 92 | 2.1 | 0.49 | 1.2 | Mild cloudy | Y | Cef 2g/dayX2 days | Full recovery | SP (37) | EBV (38) |
| 3 | 4 | Y | 4 | NA | NA | 0.6 | 0.91 | 0.68 | Clear | N |  | Moderate sequelae | SP (38) | N |
| 4 | 7 | Y | 262 | 9 | 91 | 2 | 0.60 | 0.55 | Clear | Y | Amp 4g/day X 5days | Full recovery | SS(36)/NM(39) | N |
| 5 | 10 | Y | 29 | 28 | 68 | 2.4 | 0.63 | 0.76 | Clear | NA |  | Minor sequelae | SP (37) | EBV (40) |
| 6 | 3 | Y | 9 | NA | NA | 0.8 | 0.82 | 1.64 | Clear | N |  | Full recovery | SP (39) | N |
| 7 | 5 | Y | 189 | 49 | 51 | 2.3 | 0.58 | 0.3 | Clear | Y | Cef 4g/day X2 days | Full recovery | SS (41) | N |
| 8 | 2 | Y | 440 | 12 | 88 | 3.4 | 0.68 | 0.96 | Mild cloudy | NA |  | Full recovery | SS (37) | Mumps (34) |

Note: *admission from other hospitals; Y: yes; N: no; Cef: Cefuroxime; Amp: ampicillin
